# Role of OSCAR Signaling in Osteoclastogenesis and Bone Disease

**DOI:** 10.3389/fcell.2021.641162

**Published:** 2021-04-12

**Authors:** Iva R. Nedeva, Mattia Vitale, Ari Elson, Judith A. Hoyland, Jordi Bella

**Affiliations:** ^1^Division of Cell Matrix Biology and Regenerative Medicine, Faculty of Biology, Medicine and Health, School of Biological Sciences, University of Manchester, Manchester, United Kingdom; ^2^Department of Molecular Genetics, The Weizmann Institute of Science, Rehovot, Israel

**Keywords:** osteoclastogenesis, osteoclast-associated receptor, OSCAR, bone remodeling, bone disease, collagen, cell signaling

## Abstract

Formation of mature bone-resorbing cells through osteoclastogenesis is required for the continuous remodeling and repair of bone tissue. In aging and disease this process may become aberrant, resulting in excessive bone degradation and fragility fractures. Interaction of receptor-activator of nuclear factor-κB (RANK) with its ligand RANKL activates the main signaling pathway for osteoclastogenesis. However, compelling evidence indicates that this pathway may not be sufficient for the production of mature osteoclast cells and that co-stimulatory signals may be required for both the expression of osteoclast-specific genes and the activation of osteoclasts. Osteoclast-associated receptor (OSCAR), a regulator of osteoclast differentiation, provides one such co-stimulatory pathway. This review summarizes our present knowledge of osteoclastogenesis signaling and the role of OSCAR in the normal production of bone-resorbing cells and in bone disease. Understanding the signaling mechanism through this receptor and how it contributes to the production of mature osteoclasts may offer a more specific and targeted approach for pharmacological intervention against pathological bone resorption.

## Signaling Pathways in Osteoclastogenesis

Bone tissue undergoes continuous remodeling throughout life at a rate of approximately 10% per year (Kenkre and Bassett, 2018). Such remodeling allows adequate repair of microdamage, as well as adaptation of mass, size and shape to load requirements, thereby ensuring optimal bone strength. Bone remodeling is under the strict control of an array of regulatory molecules, and is largely accomplished by the balanced activity of osteoclasts (OCLs), which resorb bone, and osteoblasts (OBLs), which lay new bone (Rucci, 2008). Several pathological states, autoimmune conditions, certain malignancies and prolonged immobilization may tip the balance in favor of increased bone resorption (Brounais et al., 2008; Iseme et al., 2017; Napoli et al., 2017; Bilezikian et al., 2019). The normal process of aging also alters the activity of OCLs and OBLs in favor of increased bone degradation (Seeman, 2019).

Loss of bone mass is more often associated with dysregulated OCL production and function rather than impaired OBL activity (Feng and Teitelbaum, 2013). Thus, implementing successful strategies for inhibition of excessive bone resorption requires thorough understanding of the mechanisms governing the proliferation, differentiation and activation of OCLs (Yavropoulou and Yovos, 2008; Park-Min, 2018). While considerable research efforts have been dedicated to the main osteoclastogenesis signaling pathway mediated through receptor activator of nuclear factor-κB (RANK), there are significant gaps in our understanding of the role of the co-stimulatory molecules in this process. Here we review briefly the key steps in OCL differentiation and summarize the current evidence relating to the role of the osteoclast-associated receptor (OSCAR).

### The Osteoclast Cell

OCLs develop from haematopoietic stem cells through a series of morphological and functional transformations that lead to the establishment of multinucleated polarized cells (Kodama and Kaito, 2020). These are able to migrate toward bone, adhere to it, and resorb the tissue beneath in a controlled manner (Bruzzaniti and Baron, 2006; Teitelbaum, 2007). OCLs express proteins that participate both in bone demineralisation and in the degradation of the demineralised organic matrix. Mutations affecting the expression or function of these proteins impair bone resorption and lead to osteopetrosis (Bruzzaniti and Baron, 2006; Teitelbaum, 2007; Sobacchi et al., 2013; Palagano et al., 2018). Table 1 summarizes data on genetic knockout mice discussed in the text below.

**Table 1:** Summary of skeleton-related phenotypes in knockout mice for genes involved in osteoclast differentiation in the order in which they are discussed in the text.

**Gene**	**Protein**	**Skeleton-related phenotypes and diseases**	**MGI IDs**	**References**
Csf1	M-CSF	Osteopetrosis, op/op mouse Abnormal bone structure, morphology and remodeling Abnormal bone and dentin mineralisation Decreased bone resorption Abnormal osteoblast morphology and differentiation Abnormal osteoclast morphology and differentiation Decreased osteoclast cell number	1856333 5305707	Yoshida et al., 1990; Naito et al., 1991, 1997; Begg et al., 1993; Harris et al., 2012; Nakamichi et al., 2013
Csf1r	M-CSFR	Osteopetrosis Abnormal bone structure, morphology, physiology and mineralisation Failure of bone resorption Abnormal osteoblast morphology Abnormal osteoclast morphology and differentiation	2181194	Dai et al., 2002, 2004; Nakamichi et al., 2013
Spi1	PU.1	Osteopetrosis Failure of tooth eruption	3717917	Tondravi et al., 1997; Houston et al., 2007
Mitf	MITF	Osteopetrosis, mi/mi mouse Osteosclerosis Abnormal bone morphology Failure of tooth eruption Abnormal osteoclast morphology and physiology	1856087 1856085	Hodgkinson et al., 1993; Nii et al., 1995; McGill et al., 2002; Steingrimsson et al., 2002
Tnfrsf11a	RANK	Osteopetrosis Abnormal bone and tooth morphology Abnormal osteoclast differentiation Decreased osteoclast cell number	1860238 2183226 3664109	Dougall et al., 1999; Li et al., 2000; Kapur et al., 2004
Tnfsf11	RANKL	Osteopetrosis Abnormal bone and tooth morphology Abnormal bone mineralisation Decreased bone resorption Abnormal osteoclast physiology Decreased osteoclast cell number Abnormal chondrocyte morphology and differentiation	1859962 2386263 5297062 5307891 5614816	Kong et al., 1999; Kim et al., 2000; Nakashima et al., 2011; Douni et al., 2012; Palmer et al., 2016
Traf6	TRAF6	Osteopetrosis Abnormal bone and tooth morphology Decreased bone resorption Abnormal osteoclast morphology and physiology	1859953 2675469	Lomaga et al., 1999; Naito et al., 1999
Nfkb1 Nfkb2	NF-κB	Osteopetrosis Abnormal bone structure and morphology Abnormal osteoclast differentiation Decreased osteoclast cell number	3852643	Franzoso et al., 1997; Iotsova et al., 1997; Yamashita et al., 2007
Fos	c-Fos	Osteopetrosis Abnormal bone and tooth morphology Decreased osteoclast cell number	2181817	Wang et al., 1992
Nfatc1	NFATc1	Osteopetrosis Abnormal bone and tooth morphology Abnormal osteoclast differentiation Decreased osteoclast cell number	3831720	Asagiri et al., 2005; Aliprantis et al., 2008
Fcer1g	FcRγ	No skeletal effects Normal bone volume and osteoclast function, size or number	1857165	Mócsai et al., 2004
Fcer1g Tyrobp	FcRγ DAP12	Osteopetrosis Abnormal bone morphology Increased bone mass Decreased bone resorption Abnormal osteoclast differentiation	3818498	Mócsai et al., 2004
Tyrobp	DAP12	Osteopetrosis Nasu-Hakola disease Abnormal bone morphology, remodeling and mineralisation Decreased bone resorption Abnormal osteoclast physiology and differentiation	2386271 2386277	Tomasello et al., 2000; Mócsai et al., 2004; Nataf et al., 2005
Btk Tec	Btk Tec	Osteopetrosis Decreased bone resorption Decreased osteoclast cell numbers	3028887	Ellmeier et al., 2000; Shinohara et al., 2008
Itgb3	Integrin β3 subunit	Osteosclerosis Increased bone thickness Abnormal osteoclast morphology and physiology Increased osteoclast cell number	2175913	McHugh et al., 2000
Oscar	Oscar	Abnormal osteoclast differentiation Reduced osteoarthritis manifestation Decreased chondrocyte apoptosis	5301430	Barrow et al., 2011; Park et al., 2020
Oscar Tyrobp	Oscar DAP12	Abnormal bone morphology Abnormal osteoclast morphology, physiology and differentiation Decreased osteoclast cell numbers	5301435	Barrow et al., 2011

*Data compiled from the Mouse Genome Informatics database (MGI) (http://www.informatics.jax.org/)*.

OCL attachment to bone is largely mediated by α_v_β_3_ integrin, which binds to matrix proteins containing arginine-glycine-aspartate (RGD) sequences such as bone sialoprotein and osteopontin. Failure of α_v_β_3_ expression results in defective cell attachment and spreading, and in deficient bone resorption (Ross and Teitelbaum, 2005; Brunner et al., 2013). Once attached, OCLs undergo cytoskeletal rearrangement that leads to the establishment of distinct apical and basolateral membrane surfaces. The basolateral surface is located distal to the bone and is enriched in Na^+^/K^+^-ATPase, transport proteins, and receptors involved in the regulation of OCL survival, differentiation and activity (discussed below). The apical surface faces the bone and forms a ruffled border through multiple, deep infoldings of the cell membrane. This ruffled appearance is due to the continuous exocytosis of secretory vesicles containing enzymes such as matrix metalloproteinase 9 (MMP-9), tartrate-resistant acid phosphatase (TRAP) or cathepsin K, which degrade the organic components of the bone. The apical surface is also rich in H^+^-ATPase, which actively secretes protons into the underlying extracellular compartment inducing acidification and demineralization of the bone area beneath the cell (Bruzzaniti and Baron, 2006). The membrane surrounding the ruffled border (sealing zone) seals off the resorption pit and creates an isolated microenvironment between cell and bone surface (Granot-Attas and Elson, 2008). OCLs adhere tightly to the bone surface through adhesion structures called podosomes, which contain an actin-rich central core and several actin-associated proteins. The region around this core contains α_v_β_3_ integrin, adaptor proteins (e.g., vinculin, paxillin, talin), Rho GTPases, and kinases such as c-Src and Pyk2 (Granot-Attas and Elson, 2008).

### The M-CSF Pathway Controls the Survival and Proliferation of OCL Precursors

Proliferation and differentiation of OCLs require two key signaling molecules which are normally expressed by stromal cells and OBLs: macrophage colony stimulating factor (M-CSF) and RANK ligand (RANKL) (Feng and Teitelbaum, 2013). Bone morphogenetic proteins (BMPs) are also emerging as key players in osteoclast homeostasis and appear to interact with the RANKL pathway in OCL differentiation and activation. The role of BMPs in OCL signaling is reviewed on a separate contribution to this Research Topic (Lademann et al., 2020) and will not be further discussed here.

M-CSF is needed during all stages of OCL development for optimal cell production and function, but the role of this cytokine is critical for the proliferation of OCL precursor cells (OCLPs). M-CSF exerts its effects through binding to its receptor M-CSFR, which is expressed on the OCLP membrane. Downstream, this leads to activation of extracellular signal regulated kinases 1 and 2 (ERK1/2) and phosphoinositide 3 kinase (PI3K), which are key regulators of the survival and proliferation of OCLPs (Feng and Teitelbaum, 2013). Accordingly, defective signaling through this pathway, such as due to inactivating mutations in the Csf1 gene or deficiency in M-CSF, results in impaired production of OCLs and leads to an osteopetrotic phenotype in mice (op/op mice, Table 1) (Yoshida et al., 1990; Dai et al., 2002). A similar phenotype is seen in mice lacking the PU.1 transcription factor (Table 1), which positively regulates the expression of M-CSFR (Tondravi et al., 1997; Houston et al., 2007).

M-CSF-dependent activation of ERK1/2 leads to stimulation of microphthalmia-associated transcription factor (MITF) which, in turn, upregulates the expression of the B-cell lymphoma-2 (Bcl-2) anti-apoptotic protein (Weilbaecher et al., 2001; McGill et al., 2002). Mice expressing a mutant MITF (mi/mi mice) or lacking Bcl-2 also exhibit an osteopetrotic phenotype (Table 1) (Hodgkinson et al., 1993; McGill et al., 2002), again demonstrating the importance of the M-CSF signaling pathway in osteoclastogenesis.

### RANK/RANKL Signaling Is Required for OCL Differentiation

RANKL, a member of the tumor necrosis factor family, is expressed by OBLs in three forms – as a transmembrane protein, as a truncated ectodomain produced by cleavage of the membrane-bound ligand, and as a secreted protein (Findlay and Atkins, 2011). Activated T cells also produce the latter form, and this could be implicated in the bone loss seen in inflammatory and autoimmune disorders such as rheumatoid arthritis (Takahashi et al., 2008; Crotti et al., 2015).

RANKL stimulates OCL differentiation through binding to RANK, a member of the tumor necrosis factor receptor (TNFR) family that is expressed on the plasma membrane of precursor cells (Feng and Teitelbaum, 2013). Deletion of the genes coding for either RANK or RANKL (Tnfrsf11a and Tnfsf11, respectively), blocks OCL production and leads to severe osteopetrosis in mice (Table 1) and in humans (Dougall et al., 1999; Kong et al., 1999; Kim et al., 2000; Li et al., 2000; Sobacchi et al., 2013; Palagano et al., 2018), indicating that RANK-RANKL signaling is critical for osteoclastogenesis.

Binding of RANKL to RANK leads to recruitment of the adaptor protein TNFR-associated factor 6 (TRAF6) (Lomaga et al., 1999; Gohda et al., 2005). The cytoplasmic tail of RANK lacks enzymatic activity, and thus interaction with TRAF6 is required for the activation of downstream pathways (Takayanagi, 2007; Kim and Kim, 2016). Other members of the TRAF family (e.g., TRAF2, 3 and 5) also bind RANK although their contribution to osteoclastogenesis appears to be limited (Takayanagi, 2007; Park et al., 2017).

**Figure 1:**
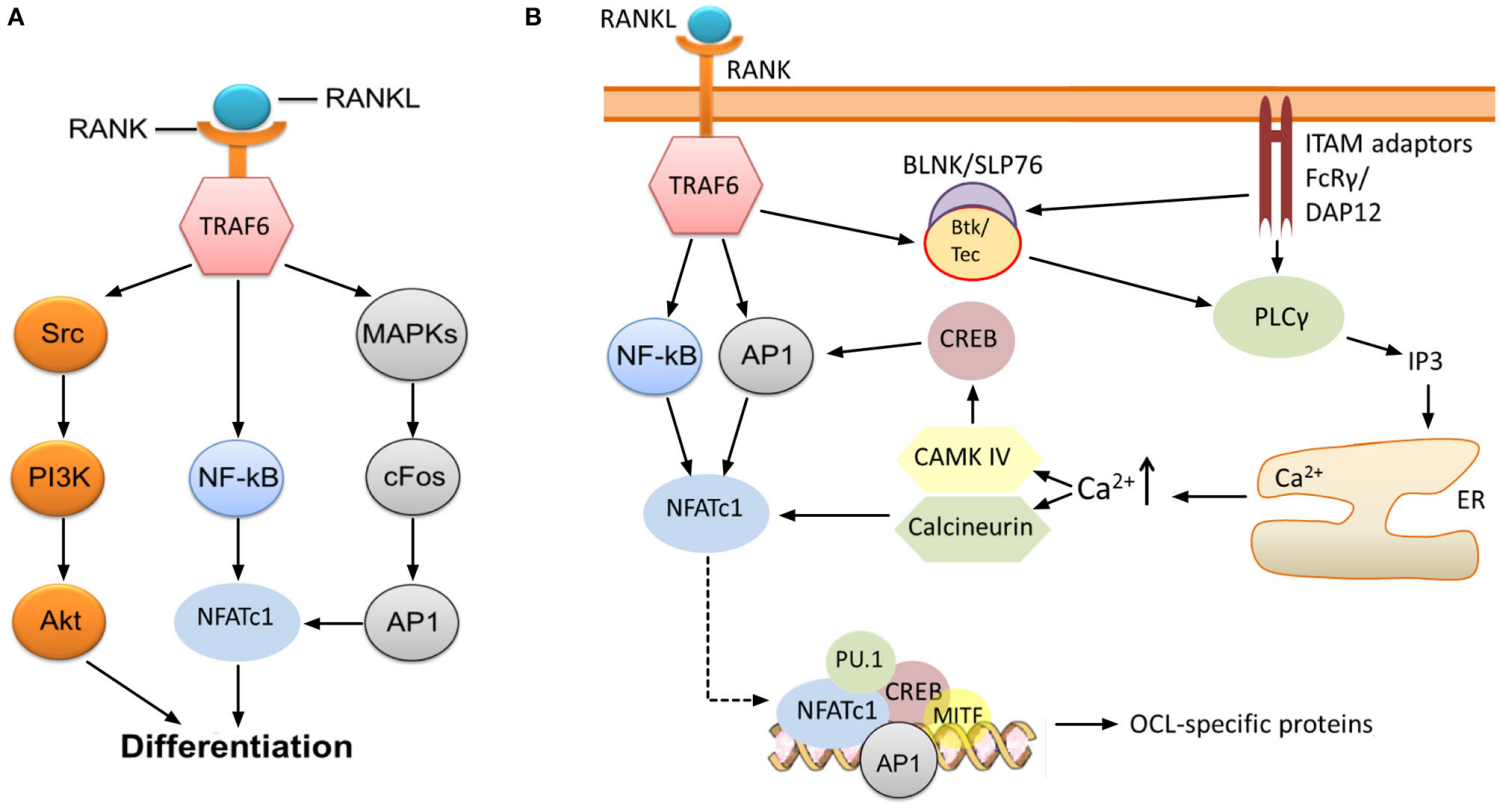
RANK-NFATc1 signaling in OCL differentiation. **(A)** Binding of RANKL to RANK leads to induction of NF-κB and AP1, which, in turn, results in increased expression of NFATc1, a key transcription factor regulating the expression of OCL-specific genes. Src-mediated activation of the PI3K/Akt pathway downstream of TRAF6 is also required for OCL production, and inhibition of these pathways impairs osteoclastogenesis. **(B)** Sustained induction of NFATc1 leads to expression of OCL-specific proteins. The initial induction of NFATc1 mediated by NF-κB and AP1 is sustained through the activities of CAMK IV and the phosphatase calcineurin. This permits robust expression of NFATc1, which, together with several other transcription factors, upregulates the expression of OCL-specific genes (see text). Signaling through FcRγ and DAP12 ITAM adaptors is required for the sustained induction of NFATc1, specifically through activation of PLCγ and subsequent release of calcium from intracellular stores. Activation of PLCγ may also need phosphorylation by Btk/Tec–BLNK/SLP76 complex which is stimulated downstream of RANK/RANKL and ITAM adaptor signaling.

RANK-recruited TRAF6 initiates signaling which ultimately leads to activation of nuclear factor kappa B (NF-κB), the kinase Akt (Wong et al., 1999; Wada et al., 2005), and several mitogen-activated protein kinases (MAPK) such as ERK1/2 (He et al., 2011), c-Jun N-terminal kinase (JNK) (David et al., 2002; Wada et al., 2005), and p38 (Li et al., 2002) (Figure 1A). One key consequence of MAPK stimulation is the induction of c-Fos and activator protein 1 (AP1), although the precise molecular mechanisms leading to this induction are not completely understood (Takayanagi, 2007; Park et al., 2017). AP1 functions as a homo/heterodimeric transcription factor, and is composed of members of the Fos, Jun and ATF (activating transcription factor) families (Hess et al., 2004). AP1/c-Fos and NF-κB directly regulate the expression of nuclear factor of activated T cells 1 (NFATc1) (Figure 1A), the key transcription factor of OCL-specific genes (Park et al., 2017). Thus, RANKL-stimulated induction of NF-κB and c-Fos is critical for the production of mature OCLs. Indeed, application of NF-κB inhibitors (Takatsuna et al., 2005) or deficiency in the NF-κB components p50/p52 (Yamashita et al., 2007) leads to impaired NFATc1 induction, while knockout of NF-κB in mice leads to osteopetrosis (Table 1) due to lack of mature OCLs (Franzoso et al., 1997; Iotsova et al., 1997). Similarly, deficiency of c-Fos abrogates NFATc1 induction and osteoclastogenesis *in vitro* (Takayanagi et al., 2002) and results in marked osteopetrosis *in vivo* (Wang et al., 1992) (Table 1), while overexpression of c-Fos can rescue NFATc1 expression in p50/p52 deficient cells (Yamashita et al., 2007).

NFATc1 upregulates the expression of TRAP, cathepsin K, calcitonin receptor, the β3 integrin subunit, DC-STAMP (dendritic cell-specific transmembrane protein) and H^+^-ATPase (Matsumoto et al., 2004; Matsuo et al., 2004; Crotti et al., 2006; Kim et al., 2008). This is accomplished through the formation of a transcriptional complex which includes NFATc1, AP1 (Fos/Jun), MITF and PU.1 (Asagiri et al., 2005) (Figure 1B), although the components of this complex may vary depending on the target gene (Takayanagi, 2007). *In vitro* studies by Takayanagi et al. (2002) showed that deletion of NFATc1 leads to failure of OCL development while ectopic expression of the protein stimulates osteoclastogenesis in the absence of RANKL. The requirement of NFATc1 for the production of OCLs was also confirmed *in vivo* (Asagiri et al., 2005; Aliprantis et al., 2008) (Table 1). However, it must be noted that global disruption of this transcription factor is lethal since its activity is similarly required for the development of cardiac valves (de la Pompa et al., 1998).

Although NF-κB and c-Fos/AP1 have critical roles for the initial expression of NFATc1, its sustained induction necessitates the activity of the calcium-calmodulin dependent protein phosphatase calcineurin and the calcium-calmodulin dependent protein kinase IV (CAMK IV) (Takayanagi, 2007). Calcineurin dephosphorylates NFATc1, which leads to exposure of its nuclear translocation signal and localization to the nucleus where NFATc1 autoamplifies its own transcription. CAMK IV activation leads to phosphorylation of cAMP response element-binding protein (CREB), and subsequently, to the robust induction of c-Fos (Takayanagi, 2007) (Figure 1B). The importance of these pathways is demonstrated by the evidence that pharmacological inhibition of calcineurin (Ishida et al., 2002; Takayanagi et al., 2002) and CAMK IV (Sato et al., 2006) results in impaired osteoclastogenesis.

Activation of calcineurin and CAMK IV depends on the rise of intracellular free calcium concentration [Ca^2+^]_i_ (Figure 1B). Although stimulation with RANKL leads to increased [Ca^2+^]_i_ (Takayanagi et al., 2002), it appears that RANK is not the receptor that directly induces changes. Indeed, there is compelling evidence indicating that the rise of [Ca^2+^]_i_ requires activation and signaling through adaptors containing immunoreceptor tyrosine-based activation motifs (ITAMs), in particular DNAX-associated protein of 12 kDa (DAP12) and FcεRI γ chain (FcRγ) (Zou and Teitelbaum, 2015; Humphrey and Nakamura, 2016) (Figure 1B).

### Co-stimulatory Signals Are Necessary for Osteoclastogenesis

The requirement for co-stimulatory signaling through DAP12 and FcRγ for OCL differentiation was demonstrated by Koga et al. (2004), who found that NFATc1 expression was nearly undetectable in DAP12^−/−^ FcRγ^−/−^ cells following stimulation with RANKL, even though c-Fos and TRAF6 were expressed. Furthermore, RANKL-induced calcium oscillations, which normally lead to activation of NFATc1, were not apparent in these cells. Similarly, phosphorylation of phospholipase Cγ (PLCγ) was found to be impaired while phosphorylation of MAPK p38 and JNK was not affected. These results demonstrate that, in the absence of the ITAM adaptors FcRγ and DAP12, pathways downstream of RANK are activated normally but the PLCγ-calcium-NFATc1 activation pathway is impaired (Figure 1B). Consequently, DAP12^−/−^ FcRγ^−/−^ OCL precursor cells fail to differentiate into mature OCLs despite the presence of RANKL and M-CSF. Ectopic expression of NFATc1 rescued OCL maturation in the DAP12^−/−^ FcRγ^−/−^ cells, while stimulation of FcRγ in DAP12^−/−^ cells restored the calcium oscillations and NFATc1 induction (Koga et al., 2004). Similarly, reintroduction of functional DAP12 in DAP12^−/−^ FcRγ^−/−^ cells rescued OCL differentiation in response to RANKL and M-CSF stimulation, whereas an ITAM-deficient DAP12 mutant failed to do so. Reintroduction of functional FcRγ was also able to rescue the osteoclastogenesis in cells doubly deficient in DAP12 and FcRγ, although this occurred only when OCL precursor cells were co-cultured with OBLs. Again, an ITAM-deficient FcRγ mutant did not rescue OCL differentiation (Koga et al., 2004). These results indicate that DAP12 and FcRγ may have overlapping roles in stimulating NFATc1 induction, although it appears that FcRγ requires a stimulus that is provided externally by OBL cells.

Similarly, Mócsai et al. (2004) observed that combined deficiency of DAP12 and FcRγ impairs osteoclastogenesis (Table 1). The precursor cells failed to form multinucleated OCLs, although they stained positive for TRAP, calcitonin receptor, cathepsin K, RANK, and integrin β_3_ (Mócsai et al., 2004). This suggests that ITAM adaptor signaling is critical for the intermediate/late stages of osteoclastogenesis, such as OCL fusion and activation. Single deficiency of DAP12 was partly compensated by FcRγ. However, this only occurred in co-culture conditions with OBLs, which is in agreement with the findings of Koga et al. (2004).

Shinohara et al. (2008) further suggested that the Tec family of kinases, specifically Btk and Tec, are required to phosphorylate and activate PLCγ in the PLCγ-calcium-NFATc1 pathway (Figure 1B). Double deficiency of Btk and Tec was associated with severe impairment of osteoclastogenesis in mice resulting in an osteopetrotic phenotype (Table 1). Furthermore, phosphorylation of PLCγ and the oscillations of calcium were suppressed in Btk^−/−^ Tec^−/−^ cells, suggesting that the kinases regulate this pathway. The authors proposed that Btk and Tec are phosphorylated in response to RANKL stimulation, following which the kinases form a signaling complex with adaptor molecules such as B-cell linker protein (BLNK) and SH2-containing leucocyte protein of 76 kDa (SLP-76). These adaptors require activation by ITAM adaptor signaling, and formation of signaling complexes was not observed in DAP12^−/−^ FcRγ^−/−^ cells. Thus, Shinohara et al. (2008) suggested that the Btk/Tec–BLNK/SLP76 complex might bridge the ITAM and RANKL pathways (Figure 1B). Nevertheless, other adaptor molecules may also be involved since double deficiency of BLNK and SLP-76 does not appear to markedly alter bone phenotype *in vivo* (although *in vitro* this is associated with significant inhibition of osteoclastogenesis) (Shinohara et al., 2008).

*In vivo* studies indicate that mice deficient in DAP12 exhibit only a mild form of osteopetrosis and have normal numbers of OCLs (Koga et al., 2004; Mócsai et al., 2004) (Table 1). Similarly, mice deficient in FcRγ appear to have normal trabecular bone volume and OCL numbers. However, mice deficient in both DAP12 and FcRγ were found to be severely osteopetrotic, and showed increased numbers and thickness of bone trabeculae (Koga et al., 2004; Mócsai et al., 2004) (Table 1). The mice had few OCLs, indicating that the observed bone changes stem from defective osteoclastogenesis. Thus, in agreement with the *in vitro* studies, these findings indicate that the ITAM adaptors are essential for OCL production *in vivo*, and that signaling through FcRγ can largely compensate for deficiency in DAP12. Indeed, Nasu-Hakola (NH) disease in humans results from deficiency of DAP12 signaling (Paloneva et al., 2000, 2002). This condition is associated with the formation of bony cysts. However, patients with NH disease show normal OCL differentiation and do not suffer from osteopetrosis, likely as a result of compensation through FcRγ. Surprisingly, these patients may even present with loss of trabecular bone (Paloneva et al., 2000), suggesting that, at least in humans, DAP12 and FcRγ may not have completely overlapping roles.

### ITAM Adaptor Signaling Is Required for OCL Activity

**Figure 2:**
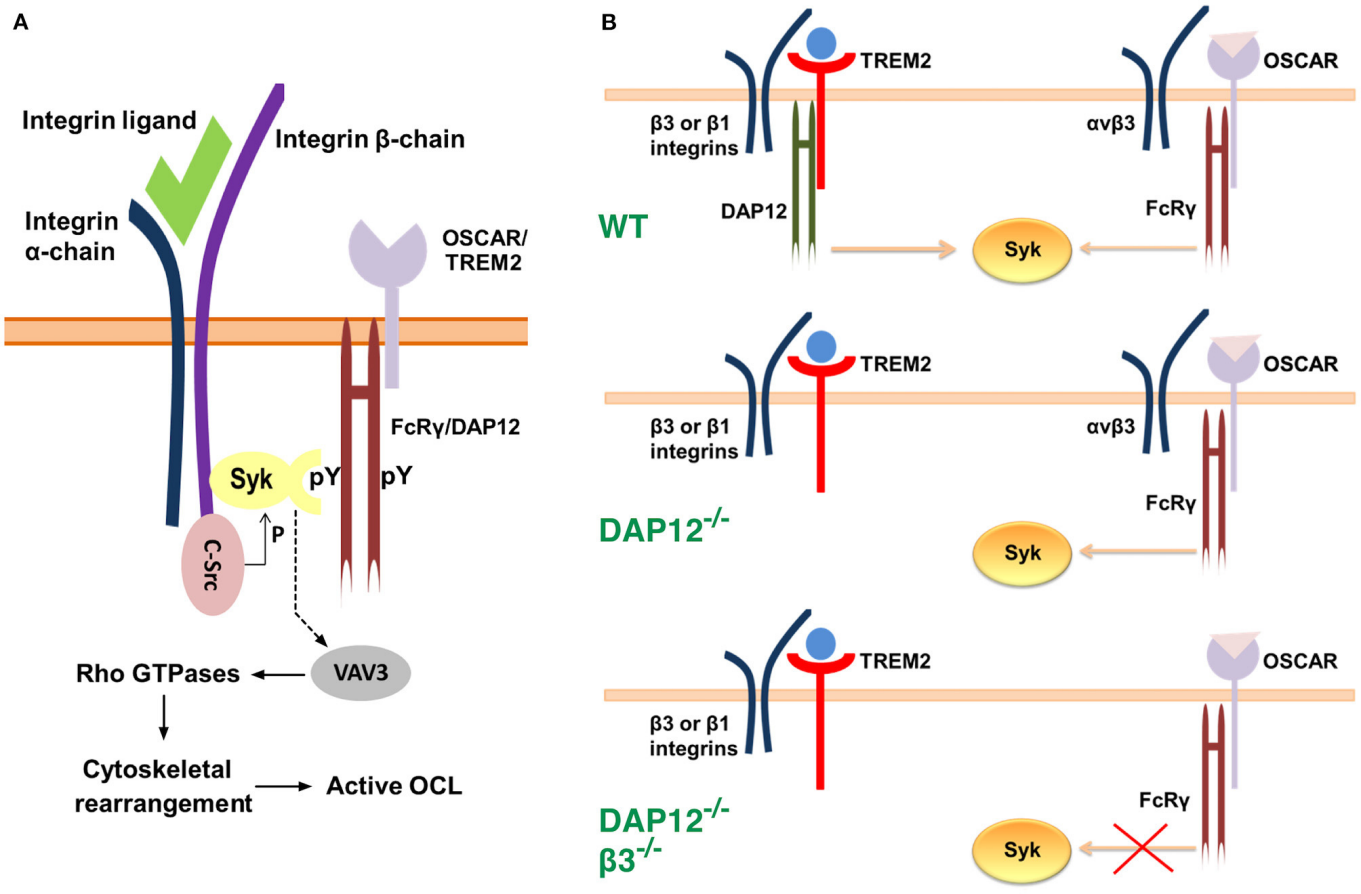
Models for the interaction between α_v_β_3_ integrins, c-Src, Syk and the ITAM adaptors. **(A)** Syk, c-Src and α_v_β_3_ integrins associate into a multimeric complex (Zou et al., 2007). c-Src is associated constitutively with the three terminal amino acids of the β_3_ integrin subunit. Binding of a ligand to α_v_β_3_ integrin leads to phosphorylation and activation of c-Src, and to the recruitment of Syk. The latter is bound to the phosphotyrosine residues of an ITAM adaptor, FcRγ or DAP12, via its C-terminal SH2 (Src homology region 2) domain. The N-terminal SH2 domain of Syk associates with the cytoplasmic domain of the β_3_ integrin subunit at a site which is distinct from that of c-Src. Activated c-Src phosphorylates Syk, which, in turn, leads to phosphorylation and activation of the guanine exchange factor Vav3. The latter alters the activity of Rho GTPases which ultimately results in reorganization of the cytoskeleton. **(B)** A model for OSCAR-FcRγ role in cytoskeletal rearrangement during osteoclastogenesis (Zou and Teitelbaum, 2015). In wild-type OCLs Syk activation is achieved mainly through TREM2-DAP12 signaling in association with β_3_ or β_1_ integrins. Syk activation leads to cytoskeletal rearrangement. Deficiency of DAP12 is compensated by OSCAR-FcRγ, which only associates with α_v_β_3_ integrin. Thus, deficiency of both DAP12 and α_v_β_3_ integrin leads to impaired signaling even in the presence of OSCAR-FcRγ and consequently to cytoskeletal disorganization.

As indicated earlier, OCLs must adhere to the bone matrix and undergo cytoskeletal rearrangement to acquire bone-resorbing activity. These processes occur during the late stages of osteoclastogenesis and require the expression of the integrin subunit β_3_ which is not present in immature OCL precursor cells (Ross and Teitelbaum, 2005). OCLs use α_v_β_3_ integrin not only to attach to the bone matrix but also to initiate signaling which leads to reorganization of the cell cytoskeleton. Research evidence suggests that FcRγ and DAP12 co-operate with α_v_β_3_ integrin in a manner that requires the activities of the c-Src and Syk kinases (Zou et al., 2007) (Figure 2). Indeed, OCLs deficient in the integrin β_3_ subunit, c-Src, or Syk fail to undergo cytoskeletal rearrangement and to form actin rings, which leads to impaired bone resorption and osteopetrosis *in vivo* (Jakus et al., 2007; Zou and Teitelbaum, 2015) (Table 1). In addition, OCLs doubly deficient in FcRγ and DAP12 have a phenotype similar to $\beta_{3}^{-/-}$ cells (McHugh et al., 2000; Faccio et al., 2003; Mócsai et al., 2004). Culture on α_v_β_3_ ligands, such as vitronectin, leads to phosphorylation and activation of Syk in OCL precursors (Faccio et al., 2003; Zou et al., 2007) while in mature adherent OCLs Syk is constitutively phosphorylated (Mócsai et al., 2004). Syk phosphorylation, however, is absent in DAP12^−/−^ FcRγ^−/−^ cells, and is attenuated in DAP12^−/−^ cells (Mócsai et al., 2004). Co-culture of OCLs with OBLs partially normalizes OCL differentiation and resorption activity in DAP12^−/−^ cells, indicating again that FcRγ can compensate for the deficiency of DAP12 in co-culture conditions (Mócsai et al., 2004).

### The ITAM Adaptor FcRγ Associates With OSCAR to Provide Co-stimulatory Signals for Osteoclastogenesis

The tyrosine-based activation motif that gives name to the ITAM adaptor molecules FcRγ and DAP12 is a conserved, short cytoplasmic sequence with a repeated signature set of four amino acids YxxI/L, typically separated by 6–8 amino acids, YxxI/Lx_(6−8)_YxxI/L (Underhill and Goodridge, 2007; Ivashkiv, 2009). Phosphorylation of the two tyrosine residues by members of the Src family of kinases allows the recruitment of signal mediators such as the Syk kinase, which ultimately alter the activity of downstream effectors and cellular activity (Merck et al., 2004; Jakus et al., 2007). The ITAM adaptors, however, do not possess an extracellular ligand-binding domain and therefore require association with specific cell-surface receptors. FcRγ has been found to associate with several immunoreceptors including OSCAR, paired immunoglobulin receptor A (PIR-A) and Fc receptors, while DAP12 has been shown to pair with triggering receptor expressed on myeloid cells 2 (TREM2), signal-regulatory protein β1 (SIRPβ1), sialic acid-binding immunoglobulin-like lectin 15 (Siglec-15), and myeloid DAP12-associated lectin (MDL-1) (Humphrey and Nakamura, 2016). The role of these receptor molecules in osteoclastogenesis is not well-understood although evidence indicates that they are involved in the provision of co-stimulatory signals (Humphrey and Nakamura, 2016). Application of activating anti-TREM2 and anti-SIRPβ1 antibodies was shown to stimulate the differentiation of OCL precursor cells, and this was only observed in the presence of DAP12 (Koga et al., 2004). Similarly, application of stimulating anti-OSCAR and anti-PIR antibodies activated osteoclastogenesis *in vitro*, and this effect was not observed in FcRγ^−/−^ OCLs (Koga et al., 2004). This evidence suggests that the ITAM adaptors may pair with more than one receptor to stimulate OCL maturation. However, it appears that receptors which pair with FcRγ do not pair with DAP12, and *vice versa*. Similar results were obtained by Merck et al. (2004), who used immunoprecipitation to demonstrate that OSCAR specifically associates with FcRγ but not with DAP12. Blockade of OSCAR signaling was found to inhibit the formation of multinucleated OCLs as well as their bone resorption activity, as evidenced by the reduction in the number of resorption pits when cells were cultured on dentine slices in the presence of a soluble form of OSCAR (Kim et al., 2002). These findings indicate that OSCAR regulates OCL differentiation in a manner which agrees with the role of its adaptor FcRγ as discussed above.

Barrow et al. (2011) examined the effect of OSCAR deficiency *in vivo* by generating DAP12^−/−^ Oscar^−/−^ mice (Table 1). These mice displayed a phenotype similar to that of DAP12^−/−^ FcRγ^−/−^ mice (Table 1). Specifically, they showed decreased OCL differentiation as indicated by the number of TRAP positive cells, as well as decreased cell size and reduced activity as evidenced by the eroded bone areas. Bone formation and OBL numbers in these mice were not different from those observed in mice deficient in DAP12 only. Consistently, the number and volume of trabeculae in DAP12^−/−^ Oscar^−/−^ mice were increased compared to those in DAP12^−/−^ mice (Barrow et al., 2011). In a different study, Zou and Teitelbaum (2015) demonstrated that activation of OSCAR with a stimulating antibody rescues the dysfunctional osteoclastogenesis in DAP12^−/−^ cells. Additionally, when cultured on bone, where natural collagen ligands for OSCAR are present (discussed later), DAP12^−/−^ OCLs were able to spread, form actin rings, and resorb bone (Zou and Teitelbaum, 2015). In agreement with the studies addressed above (section Co-stimulatory Signals are Necessary for Osteoclastogenesis), OSCAR-FcRγ rescue effects required the expression of integrin β_3_ subunit. Indeed, the authors found that mice deficient in both DAP12 and the integrin β_3_ subunit showed severe osteopetrosis with a 4-fold increase in trabecular mass. In contrast, DAP12 was found to enable cytoskeletal rearrangement via association with either integrin β_1_ or β_3_ subunits (Zou and Teitelbaum, 2015) (Figure 2B). Collectively, these studies suggest that the co-stimulatory signals for osteoclastogenesis, which are mediated through FcRγ, occur in association with OSCAR, and require the expression of β_3_ integrin subunit. As mentioned, however, the ITAM adaptor shows promiscuity and can pair with PIR-A and Fc receptors. Thus, further studies are required to establish whether these FcRγ/immunoreceptor complexes contribute to osteoclastogenesis, and whether particular conditions (e.g., injury/trauma, acute or chronic inflammation) favor signaling through a particular complex.

## A Closer Look at OSCAR

### OSCAR Discovery and Structure

OSCAR (Osteoclast Associated Ig-like Receptor) was first identified by Kim et al. (2002), who coined this name since they observed that the receptor was expressed in murine preosteoclasts and mature OCLs, but not in macrophages or dendritic cells. The novel protein was found to be an immunoglobulin type receptor and a member of the leucocyte receptor complex (LRC) (Kim et al., 2002). The human version had also been annotated as polymeric immunoglobulin receptor 3 precursor (PIGR3), but the name OSCAR has been adopted in all literature ever since. Subsequent studies revealed that OSCAR expression in bone is conserved among species, suggesting that the protein has important functions in bone homeostasis (Nemeth et al., 2011). Unlike murine OSCAR, however, the human ortholog of the receptor is expressed not only in OCLs but also in other cells of the myeloid lineage, including monocytes, macrophages and dendritic cells (Merck et al., 2004).

In humans, the OSCAR gene maps to chromosome 19q13.42 in the LRC, where it lies in close proximity to other immunoreceptor genes such as those coding for leukocyte immunoglobulin (Ig)-like receptors (LILRs) and killer cell Ig-like receptors (KIRs) (Kim et al., 2002; So et al., 2003; Merck et al., 2004; Barrow and Trowsdale, 2008). These proteins share 70% amino acid sequence homology with OSCAR, they all show an Ig-like structure, and signal through FcRγ or DAP12 adaptors (Nemeth et al., 2011). Considering this similarity, it has been suggested that OSCAR might be able to interact with major histocompatibility complex (MHC) class I molecules similarly to KIR and LILR members (Ishikawa et al., 2004).

**Figure 3:**
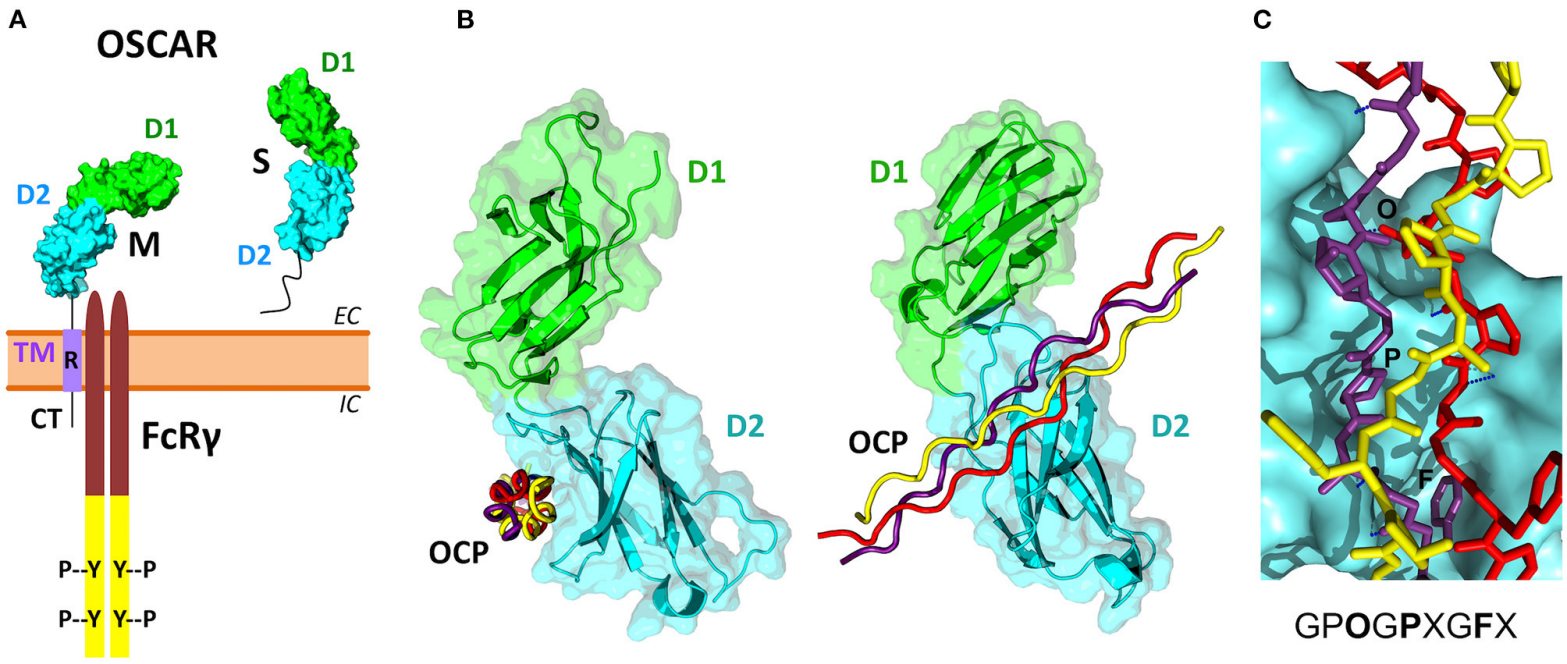
OSCAR domain structure and its association with FcRγ and collagen ligands. **(A)** Several membrane-associated (M) and soluble (S) isoforms have been reported for human OSCAR (Table 2). The M isoforms contain two extracellular Ig-like domains D1 (green) and D2 (cyan), a single transmembrane region TM (purple), and a short cytoplasmic tail CT. An arginine residue (R) within the TM region links OSCAR with the FcRγ adaptor. The S isoforms result from alternative splicing of the human OSCAR gene that removes the TM region. **(B)** Two views of the crystal structure of the OSCAR ectodomain D1-D2 in complex with an OSCAR-binding collagen-like peptide (OCP) (Haywood et al., 2016; Zhou et al., 2016). D1 and D2 are shown as ribbon and surface diagrams, and the three OCP chains are shown as red, yellow and purple ropes. **(C)** Details of the OCP bound to the primary binding site on D2. The consensus OSCAR-binding sequence is shown. Two of the three peptide chains bind tightly to the D2 surface, with several hydrogen bonds (blue dotted lines) and side chains of several residues (O, P, F) fitting D2 surface pockets.

Several isoforms have been described for human OSCAR from alternative splicing of the human gene (Table 2, Supplementary Figure 1). The OSCAR-M1 isoform is a type I transmembrane protein of 263 amino acids (245 after signal peptide removal), with two Ig-like domains D1 and D2 in its N-terminal extracellular region, a predicted transmembrane (TM) region, and a short C-terminal cytoplasmic tail (Kim et al., 2002) (Figure 3). The transcript for OSCAR-M1 contains five exons. The sequence for D1 maps to exon 3, the sequence for D2 maps to exon 4, and the sequence for the TM region is in exon 5 (Supplementary Figure 1). A longer OSCAR-S1 isoform (282 amino acids) results from the alternative splicing of exons 4 and 5 and lacks the predicted TM region (Supplementary Figure 1). Additional isoforms for human OSCAR (Table 2) arise from either adding or skipping one exon in the N-terminal region (Supplementary Figure 2). The OSCAR-S isoforms could correspond to a secreted, soluble form of OSCAR (sOSCAR, discussed later). All OSCAR-M and OSCAR-S human isoforms have identical sequences in their D1-D2 extracellular regions. Similar sets of OSCAR-M and OSCAR-S isoforms have been predicted in the genomes of chimpanzee (Table 2) and several other primates, although the state of annotation of their OSCAR genes is still preliminary.

**Table 2:** Representative entries for OSCAR genes and currently described or predicted isoforms in genome databases.

**Species**	**OSCAR gene links**	**Isoform**	**Naa**	**NCBI**	**Uniprot**
Human	ENSG00000170909	M1	263	NP_573399	Q8IYS5-2
	NCBI 126014	M2	267	NP_570127	Q8IYS5-3
		M3	252	NP_573398	Q8IYS5-6
		S1	282	NP_001269278	Q8IYS5-1
		S2	271	NP_001269279	Q8IYS5-4
		S3	286	NP_996554	Q8IYS5-7
Chimpanzee	ENSPTRG00000048476	M1	263	PNI92300	A0A2I3T010
	NCBI 107966470	M2	267	PNI92298	A0A2J8Q7Q1
		M3	252	PNI92296	A0A6D2X5E3
		S1	305	PNI92297	A0A2I3RTB0
		S2	294	PNI92302	A0A2I3TWJ1
		S3	309	PNI92299	A0A2J8Q7T4
Mouse	ENSMUSG00000054594	M1 M2	265	NP_001277306	Q8VBT3-1
	NCBI 232790	M3	271	NP_783440	Q8VBT3-2
Rat	ENSRNOG00000055716	M1	273	NP_001171902	D3ZCA1
	NCBI 292537	X1	267	XP_006228082	
Horse	ENSECAG00000039917	X2	260	XP_023506119	A0A3Q2I9K5
	NCBI 100051988	X1	292	XP_023506118	
Pig	ENSSSCG00000003259	X1	262	XP_020950544	A0A481CKH3
	NCBI 100518788				
Dog	ENSCAFG00000024209	X1	274	XP_022283292	E2RCM5
	NCBI 484313	X2	261		

*Isoform names differ in different databases. The number of amino acids (Naa) corresponds to the unprocessed sequence of each isoform*.

Two isoforms have been described for murine OSCAR (Table 2) that result from alternative splicing at the end of the first exon. Both are type I transmembrane proteins with the same domain composition as human OSCAR-M1 (Kim et al., 2002). No murine OSCAR-S type isoform has been described to date. The OSCAR gene appears to be conserved in mammals, marsupials and monotremes, and for most species only the transmembrane form is predicted. No reliable orthologs for OSCAR have been predicted to date in birds, amphibians, reptiles or fishes.

The three-dimensional structure of the extracellular region of human OSCAR has been determined by X-ray crystallography (Haywood et al., 2016; Zhou et al., 2016), both free and in complex with a collagen triple-helical peptide (discussed later) (Figure 3B). As predicted, OSCAR extracellular region consists of two Ig-like domains, D1 (membrane-distal) and D2 (membrane-proximal), connected by a short interdomain linker. The sequence of this linker diverges from the consensus linker sequence of other members of the LRC family and introduces a sharp β-turn that results an unusually obtuse interdomain angle of 241°. This obtuse angle differs significantly from the acute interdomain angles near 90° seen in other LRC receptors (Haywood et al., 2016; Zhou et al., 2016).

The cytoplasmic domain of OSCAR is short, and so far it has not been demonstrated to have a signaling function, although a putative guanylate cyclase activity has been suggested based on amino acid sequence homology analysis (Nemeth et al., 2011). In the transmembrane domain, similar to other receptors from the LRC family, OSCAR has an arginine residue at position 231 (R231) via which the receptor associates with FcRγ (Figure 3A) (Kim et al., 2002; Merck et al., 2004). This residue is conserved across OSCAR orthologs (Supplementary Figure 3). Indeed, the presence of this residue is critical for OSCAR signaling since a mutant protein in which R231 is substituted with a neutral residue fails to recruit FcRγ to the cell membrane (Merck et al., 2004).

### Regulation of OSCAR Expression

OSCAR expression appears to form a positive feedback loop with NFATc1 expression. As discussed earlier (section RANK/RANKL Signaling is Required for OCL Differentiation), RANKL stimulation in OCLs results in initial activation of NFATc1 and recruitment of other transcription factors such as PU.1 and MITF. These molecules bind to the OSCAR promoter and upregulate OSCAR expression (Kim et al., 2005b,c). Increased signaling through OSCAR-FcRγ, in turn, leads to rise in [Ca^2+^]_i_, which results in a sustained activation of NFATc1 through a Ca^2+^-dependent calcineurin pathway (Figure 1B) (Takayanagi et al., 2002), thus completing the positive feedback loop. Accordingly, application of the Ca^2+^ chelator BAPTA inhibits activation of NFATc1 in RANKL-stimulated OCLPs and suppresses osteoclastogenesis (Takayanagi et al., 2002), while application of the specific calcineurin inhibitor FK506 suppresses the increase in NFATc1 expression levels and its nuclear localization (Takayanagi et al., 2002) and markedly suppresses OSCAR expression (Kim et al., 2005c).

Several negative regulators including protein inhibitor of activated Stat3 (PIAS3), inhibitors of DNA binding (ID) proteins and the transcription factor MafB have been shown to inhibit OSCAR and NFATc1 expression and thereby to suppress RANKL-induced osteoclastogenesis (Lee et al., 2006; Kim et al., 2007a,b). PIAS3 recruits histone deacetylase 1 co-repressor to the promoters of NFATc1 and OSCAR, thus leading to arrest of their transcription. Accordingly, silencing of PIAS3 using RNAi relieves the transcriptional block and enhances osteoclastogenesis (Kim et al., 2007b). Similarly, Lee et al. (2006) observed that ID proteins interact with MITF and attenuate its ability to bind to the promoter of OSCAR. Overexpression of the ID proteins was shown to suppress the induction of OSCAR and NFATc1, and to inhibit the formation of differentiated OCLs (Lee et al., 2006).

The expression of OSCAR may be modulated by factors downstream of NFATc1. The MHC class II transactivator (CIITA), which is upregulated by NFATc1, appears to provide a negative feedback attenuating OSCAR and NFATc1 expression through competition for transcriptional binding sites (Kim et al., 2010). Modulation of MAPK signaling was also demonstrated to alter OSCAR expression. Specifically, inhibition of RANKL-induced activation of ERK, p38 and JNK by the peroxisome proliferator-activated receptor-γ (PPARγ) agonist KR62776 (Park et al., 2009) and by silibinin (Kim et al., 2009) appears to attenuate OSCAR induction and to inhibit osteoclastogenesis.

### OSCAR Ligands

When Kim et al. (2002) identified OSCAR they observed that signaling through the receptor may be bypassed when M-CSF and RANKL were supplemented externally in supraphysiological concentrations. However, signaling through OSCAR was mandatory for osteoclastogenesis when the only source of these cytokines was OBLs in a co-culture system. Application of OSCAR-Fc fusion protein, an engineered, soluble form of OSCAR fused to the Fc portion of human IgG1a, inhibited the formation of mature OCLs in co-culture conditions with OBLs (Kim et al., 2002). The authors thus proposed that OBL cells were likely expressing a putative OSCAR ligand. These findings are in agreement with studies that demonstrate that the rescue effects of FcRγ in osteoclastogenesis of DAP12^−/−^ cells are observable only when the OCL precursors are co-cultured with OBLs (Koga et al., 2004; Mócsai et al., 2004) (see section Co-stimulatory Signals are Necessary for Osteoclastogenesis). Other than type I collagen (see below), which is secreted in large amounts by OBLs, the identity of the putative OBL-expressed OSCAR ligand has not yet been confirmed. A recent review on OBL-OCL interactions summarizes the different factors involved in this cell-to-cell communication (Chen et al., 2018), some of which could indirectly affect OSCAR signaling or expression.

Nearly a decade following the discovery of OSCAR, Barrow et al. (2011) showed that collagens can serve as ligands for this receptor. They used OSCAR-Fc fusion protein and observed that it binds strongly to fibrillar collagens I, II and III. Furthermore, OSCAR-Fc did not bind to other extracellular matrix proteins such as vitronectin and fibronectin, or to collagen peptide ligands for α_2_β_1_ integrin and glycoprotein VI, indicating that OSCAR recognizes collagens containing specific sequence motifs. In agreement with the findings of Kim et al. (2002), the OSCAR-Fc fusion protein was able to bind to OBLs and stromal cells. Treatment with collagenase inhibited this interaction indicating that the putative ligands expressed on these cells are likely to contain collagenous domains (Barrow et al., 2011). Indeed, in a subsequent study Barrow et al. (2015) demonstrated that OSCAR could bind surfactant protein D (SP-D), a molecule that contains a collagen triple helical domain.

Solid-phase binding experiments with collagen toolkits, libraries of overlapping triple helical collagen-like peptides (CLPs) that encompass the entire collagen II and III sequences, elucidated the main requirements for collagen recognition by OSCAR. Barrow et al. (2011) identified the minimal OSCAR-binding collagen sequence as GPOGPXGFX (Figure 3C), where O is the abbreviation for the imino acid 4-hydroxyproline, the usual post-translational modification of proline seen in collagens and collagen-like proteins (Bella, 2016). This consensus sequence is also conserved in both chains of type I collagen. A unique motif GAOGASGDR that is quite different from the consensus sequence was found in collagen II, and several lower affinity sites were also identified (Barrow et al., 2011; Zhou et al., 2016). Furthermore, the triple-helical structure of collagen was found to be critical for OSCAR binding and signaling. Indeed, no interaction was observed when cells were cultured on an immobilized CLP containing the minimal binding collagen sequence but too short to form a triple helical structure (Barrow et al., 2011).

Human monocytes cultured on CLPs containing the minimal OSCAR-binding sequence (OCPs), were found to exhibit greater frequency of [Ca^2+^]_i_ oscillations and increased TRAP staining compared to cells cultured on control peptides (Barrow et al., 2011). In addition, murine bone marrow macrophages (BMMs) cultured on OCPs showed enhanced osteoclastogenesis as evidenced by increased gene expression of NFATc1, TRAP, cathepsin K, and the calcitonin receptor, and this response was inhibited by an anti-OSCAR antibody. Importantly, enhanced osteoclastogenesis was not observed in cells deficient in OSCAR or FcRγ, which indicates that these effects are mediated by the receptor and its adaptor molecule (Barrow et al., 2011). It is still unclear, however, whether binding of OSCAR to endogenous collagens can stimulate osteoclastogenesis in a similar manner.

Ligand-bound OSCAR was also shown to rescue osteoclastogenesis in DAP12 deficient cells (Barrow et al., 2011). When cultured on OCPs, murine DAP12^−/−^ BMMs were able to form multinucleated cells which stained positive for TRAP and exhibited well-defined podosomes. This response was absent in DAP12^−/−^ Oscar^−/−^ BMMs but was restored by retroviral transduction of OSCAR, thus indicating that the rescue effect was OSCAR-specific. The authors made similar observations when monocytes from patients with Nasu-Hakola disease (i.e., DAP12^−/−^ or TREM^−/−^) were used, thereby indicating potential clinical significance of stimulating OSCAR signaling. Altogether, the findings of Barrow et al. (2011) suggest that collagen molecules that are normally found within the extracellular bone matrix may serve as ligands for co-stimulatory signaling through OSCAR during osteoclastogenesis.

Two crystal structures of OSCAR ectodomains in complex with an OCP, published independently by Zhou et al. (2016) and Haywood et al. (2016), provided a detailed molecular view of the binding mechanism between OSCAR and its collagen ligands (Figure 3B). The structure by Zhou et al. (2016) (PDB 5EIQ) revealed two OCP binding sites on D1 and D2, respectively, while the structure by Haywood et al. (2016) (PDB 5CJB) showed a single site on D2. Site-directed mutagenesis and direct binding assays confirmed D2 as the primary binding domain. Both groups concluded that binding of collagen to D2 (membrane proximal) is facilitated by the unique angle between the two domains. Zhou et al. (2016) suggested that the interaction between OSCAR and collagen might involve two phases: an initial low-affinity binding mediated by D1, followed by a subsequent stable adhesion to D2. Notably, this contrasts with the interaction of collagen with other related receptors (such as glycoprotein VI and leukocyte-associated immunoglobulin-like receptor 1), where the D1 domain serves as a primary binding site (Lecut et al., 2004; Brondijk et al., 2010). Furthermore, Zhou et al. (2016) speculated that this two-phase interaction might be of particular importance for cells within the circulation. The authors argue that the flexibility of the D1-D2 interdomain region would permit D1 to probe the environment and mediate a weak interaction if a suitable ligand is present, which would then be followed by a firm adhesion via D2 (Zhou et al., 2016).

Haywood et al. (2016) also investigated the effects of OCP binding on osteoclastogenesis, and found that peptides containing ≥40 amino acids were able to inhibit the differentiation of cells cultured on immobilized collagen. The authors thus proposed that synthetic OCPs could potentially be used as a pharmacological treatment of pathological bone resorption (Haywood et al., 2016). Indeed, further research in this area has the potential to bring novel therapeutic strategies considering the putative role of OSCAR in bone disease as addressed in the paragraphs below.

## Physiological Significance of OSCAR in Bone Health and Disease

**Figure 4:**
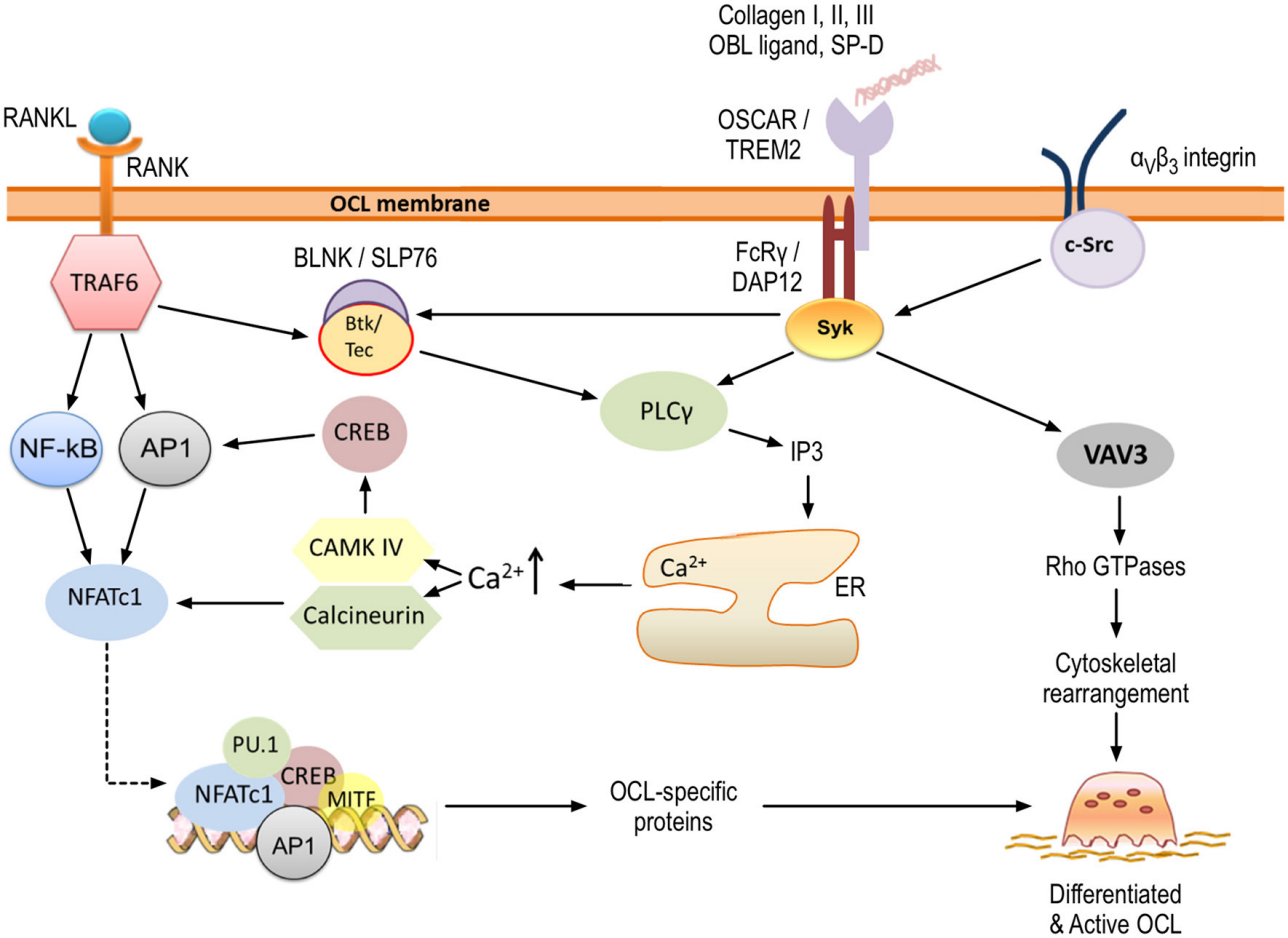
OSCAR signaling in osteoclastogenesis. OSCAR associates with FcRγ and provides co-stimulatory signals for osteoclast maturation and activation. RANK-RANKL interaction leads to initial induction of NFATc1, which is amplified through OSCAR/FcRγ-mediated activation of CAMK IV and calcineurin. Ultimately, this leads to expression of osteoclast-specific proteins. In addition, OSCAR-FcRγ, in association with α_v_β_3_ integrin, provides signals for cytoskeletal reorganization and thus osteoclast activation. Key tyrosine residues within FcRγ are phosphorylated by members of the Src family, leading to the recruitment of Syk kinase. The latter stimulates the activity of downstream effectors such as phospholipase PLCγ and guanine exchange factor VAV3, which subsequently activate further targets as shown. Details are provided within the body of the text.

Figure 4 summarizes the different signaling mechanisms addressed above and illustrates the current understanding of OSCAR contribution to signaling during osteoclastogenesis. Genetic variants for the human OSCAR gene are known and can be browsed in genome databases such as ENSEMBL (see Table 2 for the link to the human gene entry). Nevertheless, information about the possible clinical outcomes of these variants is still very limited (one exception is discussed in section OSCAR in Pathological Bone Degradation below).

### OSCAR in Bone Development, Maintenance and Repair

The interaction between OSCAR and collagen may play a significant role in the normal development, maintenance and repair of bone. During bone development OCL precursors are deposited at sites rich in collagen, and this may facilitate the production of mature OCLs through OSCAR signaling (Barrow et al., 2011). During bone maintenance and repair the recruitment of precursor cells from the circulation to bone surfaces requires transendothelial migration through blood capillaries that express RANKL and collagen III (Kindle et al., 2006; Andersen et al., 2009). It is possible that RANK-RANKL signaling and collagen-OSCAR interaction at this stage begin the process of precursor cell differentiation. Once the cells arrive at the bone surface they may encounter osteoblastic lining cells or be exposed to collagens I and III from the bone matrix (Andersen et al., 2009; Barrow et al., 2011). Further interaction between OSCAR and these ligands in the presence of RANKL may facilitate the production of multinucleated OCLs, which adhere to the bone via integrins, polarize, undergo cytoskeletal rearrangement, and begin resorption.

### OSCAR in Pathological Bone Degradation

Defective OSCAR signaling may be linked to pathological bone degradation and increased bone fragility. Kim et al. (2005a) identified 10 polymorphisms of human OSCAR, one of which was caused by a single nucleotide substitution in the promoter region (2322A>G). Using regression analysis the researchers found that this allele was strongly linked to lower bone mineral density and increased fracture risk in postmenopausal women (Kim et al., 2005a). The authors noted that the mutation might affect a putative binding site for CREB, although further research is necessary to confirm this potential mechanism.

Deregulation of ITAM signaling has been shown to contribute to the pathogenesis of inflammatory bone disease including rheumatoid arthritis, periprosthetic osteolysis, and periodontal disease (Crotti et al., 2015). Increased OSCAR and FcRγ levels were found in human periprosthetic tissue near sites of bone loss (Alias et al., 2012). Furthermore, polyethylene particles were observed to stimulate resorption by OCLs *in vitro* as well as to significantly increase the levels of OSCAR and FcRγ (Alias et al., 2012). Similarly, OSCAR has been identified at sites of osteolysis in tissues with periodontitis and in mild gingivitis, where it co-localized with TRAP-positive cells (Crotti et al., 2015). Further studies, however, are required to investigate the association of OSCAR and other ITAM-related molecules with periprosthetic bone loss and periodontitis.

### OSCAR in Rheumatoid Arthritis: Increased Expression in Mononuclear OCL Precursors

Rheumatoid arthritis (RA) is an autoimmune condition characterized by inflammation of the synovial membranes, infiltration of inflammatory cells, and increased production and activation of OCLs, which ultimately leads to bone erosion and joint destruction (Panagopoulos and Lambrou, 2018). Joints of RA patients only show mature OCLs close to the bone surface (Gravallese et al., 1998), but numerous mononuclear OCL precursors at different differentiation stages toward the OCL lineage are found in the vicinity of the joint, coming through the bloodstream and synovial microvasculature.

Not surprisingly, OSCAR has been detected in all these cells. Herman et al. (2008) showed that OSCAR is strongly expressed in the synovial tissue of RA patients. Staining of RA specimens revealed that OSCAR is expressed by multinucleated OCLs at the bone surface and approximately by 30% of mononuclear cells around synovial microvessels. Furthermore, circulating peripheral monocytes from RA patients showed 2-fold higher expression of OSCAR than monocytes from healthy individuals, suggesting that the receptor expression is increased before the cells enter the synovial tissue (Herman et al., 2008). *In vitro* culture demonstrated that these monocytes exhibited significantly higher differentiation into OCLs than cells with lower OSCAR levels from healthy individuals. Addition of OSCAR-Fc fusion protein to the culture inhibited the enhanced osteoclastogenesis and the effect was dose-dependent, reflecting competition for OSCAR ligand. These data suggest that the OSCAR pathway is activated in monocytes of RA patients, potentially exacerbating OCL differentiation and bone resorption (Herman et al., 2008). Similarly, Crotti et al. (2012) found that OSCAR and FcRγ were expressed in macrophage-like cells from the synovial tissue of active RA, and their levels were higher than those in tissue of inactive RA or healthy individuals. TREM2 and DAP12 were also found to be upregulated in inflammatory bone disease (Crotti et al., 2012; Chen et al., 2014). These results suggest that the enhanced destruction of bone seen in RA may partly be due to OSCAR-mediated differentiation of monocytes into OCLs, and further research is required to understand the relative contribution of TREM2-DAP12 and OSCAR-FcRγ complexes to bone disease.

### OSCAR Beyond OCLs: Excessive Activation of the Immune System

Excessive and inappropriate activation of the immune system presents another mechanism via which OSCAR may contribute to the pathology of RA. Several studies have shown that human OSCAR is expressed not only in monocytes but also in other cells of the myeloid lineage, including macrophages, neutrophils and dendritic cells (Merck et al., 2004, 2005, 2006). Similarly to the signaling events in OCLs, activation of OSCAR in these cells was associated with an increase in [Ca^2+^]_i_ mediated through the ITAM-containing adaptor FcRγ. Dendritic cells (DCs) were found to express OSCAR continuously during all stages of differentiation, and the receptor was also maintained on the surface of mature cells (Merck et al., 2004). Ligation of OSCAR with an anti-OSCAR monoclonal antibody was shown to result in endocytosis of the receptor in a manner similar to the anti-mannose receptor internalization. The OSCAR-antibody complex was transported to vesicles associated with MHC class II-mediated antigen presentation. These findings indicate that in DCs OSCAR may be involved in antigen uptake and presentation (Merck et al., 2004). Furthermore, activation of OSCAR was shown to promote DC survival in a manner dependent on ERK and PI3K-induced expression of the anti-apoptotic proteins Bcl-2 and Bcl-xL (Merck et al., 2005). Stimulation of OSCAR was associated with increased maturation of DCs as well as with increased secretion of cytokines and chemokines such as the interleukins IL-8 and IL-12p40, monocyte chemoattractant protein-1 (MCP-1) and macrophage-derived chemokine (MDC). In addition, OSCAR signaling enhanced the stimulatory effects of Toll-like receptor ligands on DC maturation, cytokine release, and ability to induce T-cell proliferation and activation (Merck et al., 2005).

Following the identification of collagens as OSCAR ligands Schultz et al. (2015) demonstrated that collagen types I and II bind to OSCAR expressed on the surface of human DCs. Similar to the findings of Merck et al. (2004, 2005), the collagen-OSCAR interaction promoted the survival of DCs and enhanced the secretion of chemokines and proinflammatory cytokines, including tumor necrosis factor α (TNF-α), IL-6, IL-8, IL-10, IL-13, IL-23, and RANTES (Regulated on Activation, Normal T Cell Expressed and Secreted chemokine). Cells cultured on collagens I and II showed enhanced expression of maturation markers such as CD86 and CD83, and this effect was inhibited in a dose-dependent manner by the addition of OSCAR-blocking antibody (Schultz et al., 2015). These findings indicate that collagen-OSCAR interaction promotes DC maturation and activity, including secretion of pro-inflammatory cytokines, antigen uptake and induction of T lymphocytes.

Similar to the findings presented above, OSCAR signaling in monocytes was found to upregulate the expression of adhesion molecules and to increase the secretion of cytokines and chemokines including IL-8, MCP-1, MDC and ENA-78 (epithelial neutrophil-activating peptide-78) (Merck et al., 2006). The monocytes showed an increased lifespan when cultured in the absence of pro-survival factors. In contrast, OSCAR signaling did not affect the survival of neutrophils. However, the cells showed enhanced degranulation, synthesis of reactive oxygen species and cytokine release. Activation of OSCAR in neutrophils was also associated with changes in the expression of cell surface molecules involved in their recruitment (Merck et al., 2006). Collectively, these studies demonstrate that OSCAR signaling affects the function of both the innate and adaptive immune system, and thus it may play a role in inflammatory-mediated bone loss not only through supporting osteoclastogenesis but also through the enhanced recruitment and activation of immune cells.

Furthermore, a positive feedback loop may exist between OSCAR expression and immune cell activation in RA. As mentioned above, levels of OSCAR are higher in the synovial tissue of active RA compared to inactive RA (Crotti et al., 2012), suggesting that expression of OSCAR may be regulated by inflammatory cytokines. Indeed, Herman et al. (2008) investigated the effects of TNFα on OSCAR expression in monocytes *in vitro* and found that OSCAR mRNA was increased 4-fold in response to the treatment. OSCAR expression was also found to correlate with disease severity as indicated by the levels of C-reactive protein and erythrocyte sedimentation rate (which are markers of systemic inflammation), as well as by clinical assessment of RA activity (Herman et al., 2008).

Crotti et al. (2012) observed high OSCAR expression in the microvasculature of synovial tissue from RA patients and none in healthy control samples. OSCAR was detected on the luminal side of the microvasculature, suggesting association with endothelial cells. The authors hypothesized that the receptor is either expressed by endothelial cells or produced and secreted by other cells, following which it becomes bound to the endothelium at sites of inflammation (Crotti et al., 2012). Indeed, an earlier study by Goettsch et al. (2011) identified OSCAR in primary endothelial cells where its expression was found to be regulated by oxidized low density lipoprotein (oxLDL) via lectin-like oxidized LDL receptor 1 (LOX-1). The oxLDL-LOX1 interaction stimulated OSCAR induction in a manner that was dependent on activation of PI3K, calcium signaling and NFATc1. Similar to the anti-apoptotic effects of OSCAR in DCs and monocytes, stimulation of the receptor enhanced the survival of endothelial cells cultured in serum-free conditions (Goettsch et al., 2011). In a further study, the researchers found that OSCAR was upregulated in monocytes and macrophages from ApoE knockout mice fed on high-fat diet (Sinningen et al., 2013). TNFα and oxLDL produced an increase in OSCAR mRNA levels in RAW264.7 cells, while blockade of LOX-1 and NF-κB pathway abolished these effects (Sinningen et al., 2013). These studies, thereby, indicate that OSCAR expression is upregulated in endothelial cells and macrophages in response to pro-inflammatory stimuli.

Interestingly, changes in the level of OSCAR expression during bone disease may not be completely mirrored by those of FcRγ. Crotti et al. (2012) detected OSCAR but not FcRγ in the microvasculature of RA patients. In another study by Andersson et al. (2007), mouse calvarial bones cultured with synovial fluid from patients with osteoarthritis showed enhanced OSCAR and NFATc1 mRNA expression, while FcRγ levels remained unchanged relative to control. Considering this evidence, it may be speculated that in disease states OSCAR signaling may become uncoupled from FcRγ. As indicated earlier (section OSCAR Discovery and Structure) the cytoplasmic tail of OSCAR contains an amino acid sequence which suggests a potential guanylate cyclase activity (Nemeth et al., 2011). Furthermore, OSCAR has been observed on the OCL surface in the absence of FcRγ, albeit the presence of the adaptor molecule was found to upregulate the receptor expression (Ishikawa et al., 2004). Thus, signaling through OSCAR independently of FcRγ may be possible, although evidence for this is lacking at present.

### OSCAR Expression in Chondrocytes: A Link to Osteoarthritis

Osteoarthritis (OA) has often been described as a degenerative joint disorder where the articular cartilage breaks down as a result of wear and tear, causing the bones to rub against each other, producing pain. However, OA is better defined as a whole joint disease that also affects subchondral bone, synovial membrane, ligaments and menisci (Loeser et al., 2012; Peng et al., 2021). Development of OA involves abnormal behavior of chondrocytes and altered metabolic homeostasis of the cartilage extracellular matrix. These result in chronic inflammation and defective remodeling of articular cartilage and neighboring bone driven by matrix degrading enzymes and inflammatory cytokines (Kapoor et al., 2011; Loeser et al., 2012; Hu and Ecker, 2021; Peng et al., 2021).

A recent study has reported for the first time a clear link between OA and OSCAR (Park et al., 2020). The authors demonstrated OSCAR expression in chondrocytes of wild-type mice, indicating that cells outside the non-myeloid lineage can also produce OSCAR. Furthermore, levels of expression were markedly elevated during OA pathogenesis in mouse and human articular cartilages. Genetic deletion of OSCAR in Oscar^−/−^ mice (Table 1) reduced all hallmarks of OA pathology in an experimental model of induced OA. The study also demonstrated that OSCAR induces chondrocyte apoptosis during OA pathogenesis through its interaction with collagen, and that addition of a soluble OSCAR-Fc fusion protein attenuates OA pathogenesis in mouse models. Gene expression analysis identified a link between OSCAR and tumor necrosis factor-related apoptosis-inducing ligand (TRAIL), coded by the Tnfsf10 gene in mice and responsible for chondrocyte apoptosis in OA pathogenesis. TRAIL expression was downregulated in OA chondrocytes in Oscar^−/−^ mice and in OA mice treated with OSCAR-Fc fusion protein. Thus, there seems to be an association between the interaction of OSCAR with collagen and the levels of TRAIL expression in the development and progression of OA. Park et al. (2020) conclude that disruption of the OSCAR–collagen interaction is therefore a new avenue for the development of small molecule inhibitors or biologics as therapeutic agents against OA.

### A Secreted, Soluble Form of OSCAR in Serum

The discrepancy between the expression levels of OSCAR and FcRγ seen in diseased bone tissue may in part be associated with the detection of a secreted form of OSCAR. Indeed, a soluble form of OSCAR (sOSCAR) was initially identified in human blood serum (Herman et al., 2008), following which it was detected in the synovial fluid from RA patients (Crotti et al., 2012). Serum levels of sOSCAR were found to be decreased in RA patients compared to healthy individuals, and in a manner which correlated inversely with disease severity (Herman et al., 2008; Zhao et al., 2011). Furthermore, anti-TNFα therapy was demonstrated to significantly increase the levels of sOSCAR in serum (Herman et al., 2008). Given this evidence it may be hypothesized that in health OSCAR is either secreted or cleaved from the membrane surface after a certain signaling threshold is reached. The sOSCAR may bind pro-osteoclastogenic ligands and serve as a decoy receptor, thus limiting excessive production of OCLs and bone resorption. In inflammatory disorders, such as RA, pro-inflammatory mediators may lead to inhibition of OSCAR secretion/cleavage, which subsequently may result in decreased serum levels of sOSCAR and sustained pro-osteoclastogenic signaling.

It must be noted, however, that conflicting evidence has also been presented, indicating that sOSCAR levels may actually be positively regulated by pro-inflammatory cytokines. Crotti et al. (2012) cultured bone marrow endothelial cells in the presence of TNFα and IL-1β, and observed increased levels of OSCAR mRNA as well as the membrane-bound and secreted forms of the protein. In addition, Ndongo-Thiam et al. (2014) reported higher levels of sOSCAR in plasma from RA patients compared to healthy individuals. The authors concluded that levels of serum sOSCAR directly correlate with disease severity and may be predictive of bone destruction. A soluble form of OSCAR has also been detected in the synovial fluid of OA and RA patients with no significant difference between these groups (Crotti et al., 2012). It is not clear, however, whether and how levels of sOSCAR in serum and in synovial fluid correlate.

Considering the findings presented here, further research is imperative to resolve the discrepancy in the existing evidence and to clarify whether the soluble form of OSCAR is positively or negatively regulated by pro-inflammatory cytokines. In addition, further studies are required to identify the exact role and mechanism of sOSCAR production. It is unclear at present whether OSCAR is cleaved from the membrane and if so what signals regulate this process. Alternatively, sOSCAR may be produced as OSCAR-S via alternative splicing, and become exocytosed immediately following translation rather than being shed from the membrane. Answering these unknowns would help to understand better the role of OSCAR in physiological and pathological osteoclastogenesis, and may lead to the discovery of new therapeutics targeting excessive bone resorption.

## Concluding Remarks

**Figure 5:**
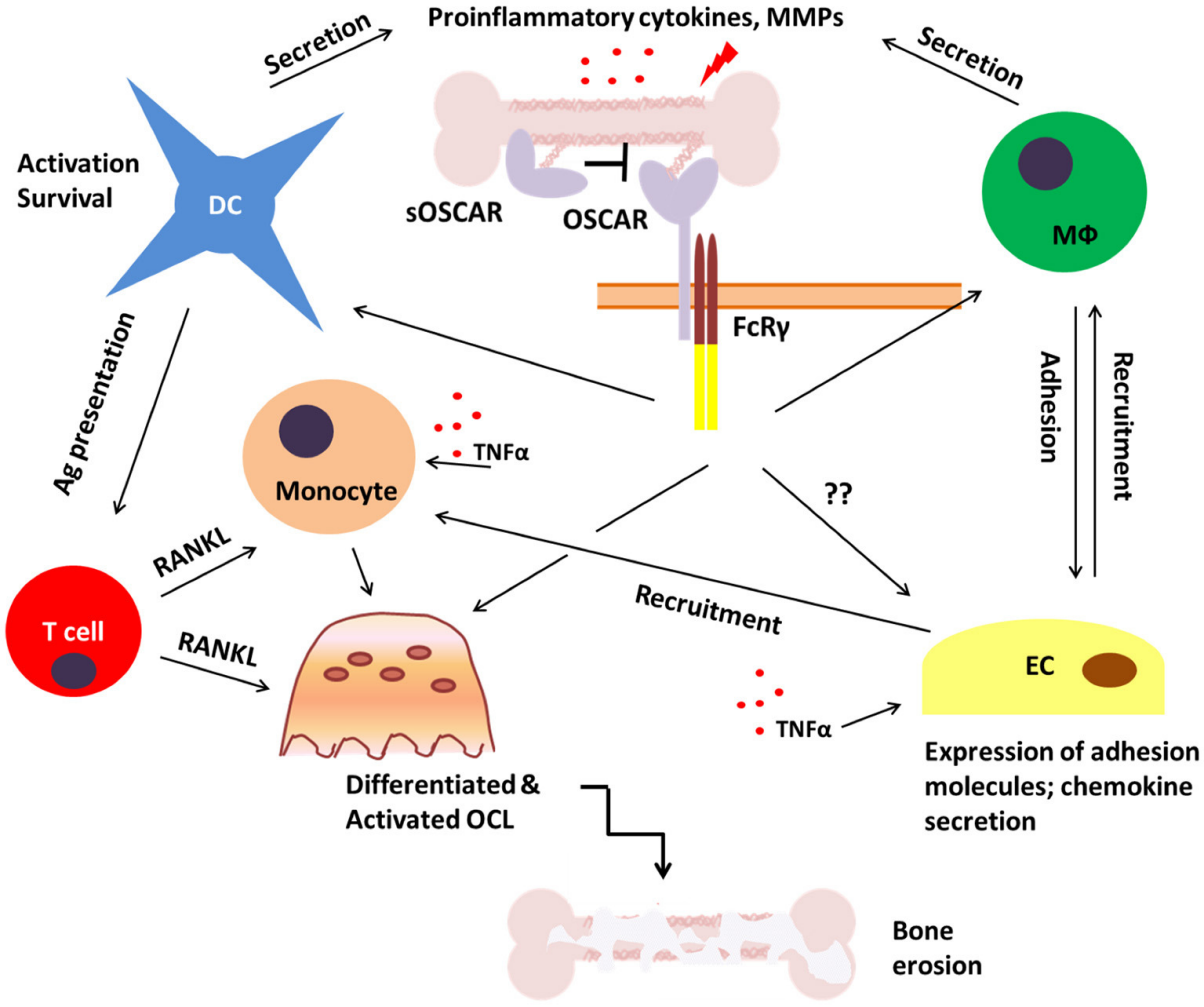
Potential role of OSCAR in bone disease. During inflammatory bone disease inappropriate and prolonged activation of immune cells may lead to enhanced release of pro-inflammatory mediators such as cytokines, chemokines and matrix metalloproteinases (MMPs). This would stimulate the expression of adhesion molecules on endothelial cells (EC) and facilitate the recruitment of OSCAR-expressing immune cells including monocytes, dendritic cells (DC), and macrophages (Mϕ). This may lead to both amplification of the inflammatory process and enhanced osteoclastogenesis which would eventually result in bone erosion and fragility. A soluble form of OSCAR (sOSCAR) may prevent excessive signaling via OSCAR-FcRγ through competition for OSCAR ligands, although the current evidence for this is conflicting. OSCAR-FcRγ complex is also expressed on ECs, but little is known about its role in these cells in relation to bone disease.

OSCAR provides co-stimulatory signals which are required for osteoclast differentiation and activation (Figure 4). Furthermore, the expression of OSCAR and its adaptor molecule, FcRγ, is upregulated in inflammatory bone disease and osteoporosis (Figure 5). Given the available evidence it is plausible to consider that modulation of the signaling through OSCAR has the potential to suppress excessive osteoclastogenesis and bone resorption. Nevertheless, although this receptor has been identified nearly 20 years ago, many unanswered questions remain. For instance, what is the relative contribution of OSCAR-FcRγ complex to osteoclastogenesis considering that the FcRγ adaptor may couple with other immunoreceptors, or that other co-stimulatory molecules such as TREM2-DAP12 are also expressed by osteoclasts? Could OSCAR have an intrinsic signaling activity (i.e., independent of FcRγ) under certain conditions? What is the identity of the ligand expressed on osteoblast/stromal cells which activates OSCAR signaling? Could certain synthetic collagen-like peptides be used to inhibit the signaling through OSCAR-FcRγ complex? What signals are involved in termination of OSCAR-FcRγ activity? Is the soluble form of OSCAR produced by secretion or cleavage, and what are the signals that regulate its release? Do serum levels of sOSCAR increase or decrease during inflammatory bone disease, and could sOSCAR serve as a diagnostic marker?

Taking into consideration that OSCAR is expressed not only on osteoclasts but also on immune and other cell types such as endothelium, it is important to better understand the signaling through this receptor, and whether it contributes to chronic inflammatory disease. Since inflammation is at the core of many disorders affecting not only bone but also other body systems, modulation of OSCAR signaling may present new opportunities for alleviating disease activity and progression.

## Author Contributions

IRN, MV, AE, JAH, and JB contributed to the literature research, discussion, and interpretation. IRN and JB drafted the manuscript. All the authors critically read, revised, and approved the final version of the manuscript.

## Conflict of Interest

The authors declare that the research was conducted in the absence of any commercial or financial relationships that could be construed as a potential conflict of interest.
