# Supplementary material for: Role of OSCAR Signaling in Osteoclastogenesis and Bone Disease
Source: Front Cell Dev Biol. 2021 Apr 12;9:641162. doi: 10.3389/fcell.2021.641162 (PMC8072347; doi:10.3389/fcell.2021.641162)
Supplement: Supplementary file 1 [file Image_1.PDF]

Supplementary Material

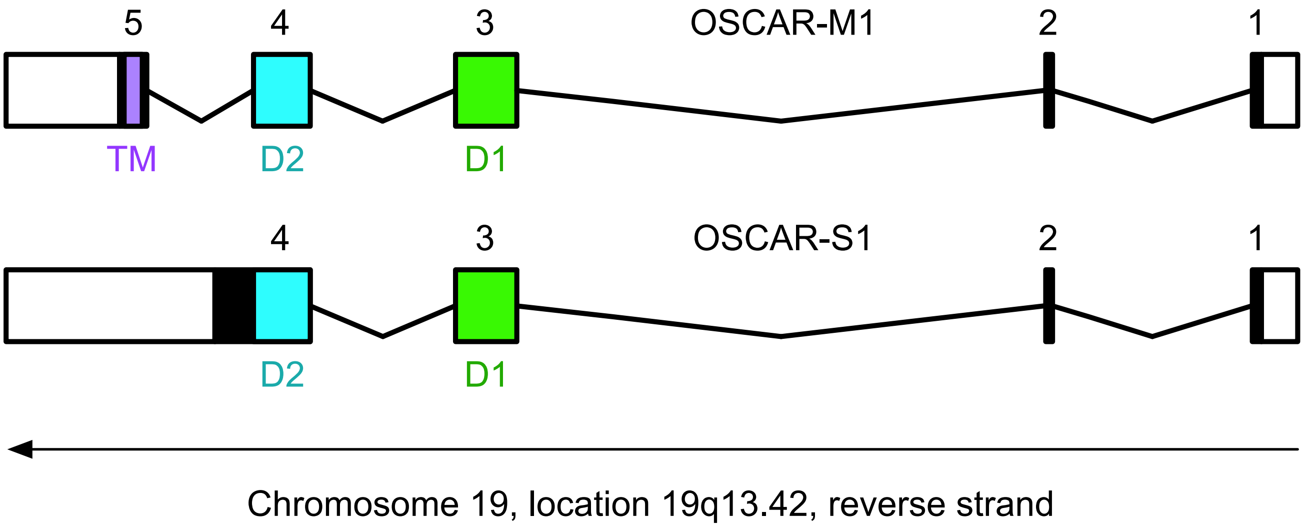

Supplementary Figure 1. Representative splice variants described in the human OSCAR gene. Exons are represented as square boxes connected by introns, and are numbered from right to left as the OSCAR gene is in the reverse strand. Filled boxes represent protein coding regions and empty boxes correspond to untranslated regions. The extracellular domains D1 (green) and D2 (cyan) map to exons 3 and 4, respectively. Two isoforms result from alternative splicing of the last two exons. OSCAR-M1 has five protein coding exons and a predicted transmembrane region (TM, purple) in exon 5. This isoform corresponds to the membrane-bound, OSCAR receptor form. OSCAR-S1 has only four protein coding exons due to intron retention. An in-frame stop codon in the retained intron means that this isoform lacks the TM region and it could correspond to a soluble form of OSCAR (see main text). Additional isoforms have been described where either exon 2 is skipped (in M3 and S2) or an additional small exon is included between exons 2 and 3 (in M2 and S3) (see Supplementary Figure 2).

```
M1|NP_573399         MALVLILQLLTLWPLCHTDITP----SVPPASYHPKPWLGAQPATVVTPGVNVTLRCRAP
M2|NP_570127         MALVLILQLLTLWPLCHTDITPSVAIIVPPASYHPKPWLGAQPATVVTPGVNVTLRCRAP
M3|NP_573398         MALVLILQLLTLF---------------PPASYHPKPWLGAQPATVVTPGVNVTLRCRAP
S1|NP_001269278      MALVLILQLLTLWPLCHTDITP----SVPPASYHPKPWLGAQPATVVTPGVNVTLRCRAP
S2|NP_001269279      MALVLILQLLTLF---------------PPASYHPKPWLGAQPATVVTPGVNVTLRCRAP
S3|NP_996554         MALVLILQLLTLWPLCHTDITPSVAIIVPPASYHPKPWLGAQPATVVTPGVNVTLRCRAP
                     ************:               ********************************

M1|NP_573399         QPAWRFGLFKPGEIAPLLFRDVSSELAEFFLEEVTPAQGGSYRCCYRRPDWGPGVWSQPS
M2|NP_570127         QPAWRFGLFKPGEIAPLLFRDVSSELAEFFLEEVTPAQGGSYRCCYRRPDWGPGVWSQPS
M3|NP_573398         QPAWRFGLFKPGEIAPLLFRDVSSELAEFFLEEVTPAQGGSYRCCYRRPDWGPGVWSQPS
S1|NP_001269278      QPAWRFGLFKPGEIAPLLFRDVSSELAEFFLEEVTPAQGGSYRCCYRRPDWGPGVWSQPS
S2|NP_001269279      QPAWRFGLFKPGEIAPLLFRDVSSELAEFFLEEVTPAQGGSYRCCYRRPDWGPGVWSQPS
S3|NP_996554         QPAWRFGLFKPGEIAPLLFRDVSSELAEFFLEEVTPAQGGSYRCCYRRPDWGPGVWSQPS
                     ************************************************************

M1|NP_573399         DVLELLVTEELPRPSLVALPGPVVGPGANVSLRCAGRLRNMSFVLYREGVAAPLQYRHSA
M2|NP_570127         DVLELLVTEELPRPSLVALPGPVVGPGANVSLRCAGRLRNMSFVLYREGVAAPLQYRHSA
M3|NP_573398         DVLELLVTEELPRPSLVALPGPVVGPGANVSLRCAGRLRNMSFVLYREGVAAPLQYRHSA
S1|NP_001269278      DVLELLVTEELPRPSLVALPGPVVGPGANVSLRCAGRLRNMSFVLYREGVAAPLQYRHSA
S2|NP_001269279      DVLELLVTEELPRPSLVALPGPVVGPGANVSLRCAGRLRNMSFVLYREGVAAPLQYRHSA
S3|NP_996554         DVLELLVTEELPRPSLVALPGPVVGPGANVSLRCAGRLRNMSFVLYREGVAAPLQYRHSA
                     ************************************************************

M1|NP_573399         QPWADFTLLGARAPGTYSCYYHTPSAPYVLSQRSEVLVISWEDSGSSDYTRGN-------
M2|NP_570127         QPWADFTLLGARAPGTYSCYYHTPSAPYVLSQRSEVLVISWEDSGSSDYTRGN-------
M3|NP_573398         QPWADFTLLGARAPGTYSCYYHTPSAPYVLSQRSEVLVISWEDSGSSDYTRGN-------
S1|NP_001269278      QPWADFTLLGARAPGTYSCYYHTPSAPYVLSQRSEVLVISWEGEGPEARPASSAPGMQAP
S2|NP_001269279      QPWADFTLLGARAPGTYSCYYHTPSAPYVLSQRSEVLVISWEGEGPEARPASSAPGMQAP
S3|NP_996554         QPWADFTLLGARAPGTYSCYYHTPSAPYVLSQRSEVLVISWEGEGPEARPASSAPGMQAP
                     ******************************************..*..  . ..

M1|NP_573399         -------------LVRLGLAGLVLISLGALVTFDWRSQNRAPAGIRP
M2|NP_570127         -------------LVRLGLAGLVLISLGALVTFDWRSQNRAPAGIRP
M3|NP_573398         -------------LVRLGLAGLVLISLGALVTFDWRSQNRAPAGIRP
S1|NP_001269278      GPPPSDPGAQAPSLSSFRPRGLVLQPLLPQTQDSWDPAPPPSDPGV-
S2|NP_001269279      GPPPSDPGAQAPSLSSFRPRGLVLQPLLPQTQDSWDPAPPPSDPGV-
S3|NP_996554         GPPPSDPGAQAPSLSSFRPRGLVLQPLLPQTQDSWDPAPPPSDPGV-
                                  *. .   **** .*  ..  .* .    ..
```

Supplementary Figure 2. Sequences of human OSCAR isoforms. Multiple sequence alignment of six described isoforms for the human OSCAR gene. NCBI entry codes for each isoform are shown. Isoforms M1-M3 have a predicted C-terminal transmembrane region (purple), which is missing in isoforms S1-S3. The sequences of the extracellular domains D1 (green) and D2 (cyan) are identical for all six isoforms. The predicted signal peptide sequence is shown in gray. Asterisks (*) indicate a fully conserved position; colons (:) and dots (.) indicate a partially conserved position.

```
M1|HUMAN           MALVLILQLLTLWPLCHTDITPSVPPASYHPKPWLGAQPATVVTPGVNVTLRCRAPQPAW
M1|CHIMPANZEE      MALVLILQLLTLWPLCHTDITPSVPPASYHPKPWLGAQPATVVTPGVNVTLRCRAPQPAW
X2|HORSE           MALVLLLQLLSLWRLCHTYTTPTVPPALYP-KPRLGAQPAAVVTPGVNVTLRCRAPLPAW
X1|PIG             MALVLLLQLLTVWPACRADITPTVPPASYP-KPWLEAQPAAIVTPGINITLRCWAPQPAW
X1|DOG             MALALILELLILWPLCHSDITPTVSPALYP-KPWLEAQPAAIVTPGVNVTLRCQAPQLAW
M2|MOUSE           MVLSLILQLSTLWPACRADFTPTAPLASYP-QPWLGAHPAAVVTPGINVTLTCRAPQSAW
X1|RAT             MVLLLILQLSTLWPVCHADFTSPVPLASYP-KPWLGAHPAAIVTPGINVTLICRAPQPAW
                   *.* *:*:*  :*  *::  *.... * *  :* * *:**::****:*:** * **  **

M1|HUMAN           RFGLFKPGEIAPLLFRDVSSELAEFFLEEVTPAQGGSYRCCYRRPDWGPGVWSQPSDVLE
M1|CHIMPANZEE      RFGLFKPGEIAPLLFRDVSSELAEFFLEEVTPAQGGSYRCCYRRPDWGPGVWSQPSDALE
X2|HORSE           RFELFKSGEIESILQRDVFLELAEFFLEEVTTEQGGSYRCCYKKRGWRPGVCSQLSDALE
X1|PIG             RFALFKYGDAAPVIYRDVASELAEFFLEEVTPAQGGSYRCCYRRLSWGPGVWSHPSDTLE
X1|DOG             RFALFKSGEITPVLYRDVSMELAEFFLEEVTPAQGGSYHCCYRSLGWDLGIWSHPSDTLE
M2|MOUSE           RFALFKSGLVTPLLLRDVSVELAEFFLEEVTPAQGGSYHCRYRKTDWGPGVWSQPSNVLE
X1|RAT             GFGLFKTGLATPLLLRNVSIGLAEFFLEKVTSVQEGSYHCRYRKTDWGPGVWSQPSNALE
                          * *** *   .:: *:*   *******:**. * ***:* *:  .*  *: *:
*:.**

M1|HUMAN           LLVTEELPRPSLVALPGPVVGPGANVSLRCAGRLRNMSFVLYREGVAAPLQYRHSAQPWA
M1|CHIMPANZEE      LLVTEELPRPSLVALPGPVVAPGANVSLRCAGRLRNMSFALYREGVAAPLQYRHSAQPWA
X2|HORSE           LLVTDELPAPTLVALPGPVVAPGANVSLRCAGRWRGMSFALYREGEAAPVQYRDSPQPWA
X1|PIG             LLVTDELPRPSLVALPGPVVAPWANVSLRCAGRVGGMSFALYRVGVAAPLQYRRSAQPWA
X1|DOG             LLVTDQLPRPSLVALPGPVVAPDANVSLRCAGRLRGMSFALYRVGVAAPLQYRDSAEPWA
M2|MOUSE           LLVTDQLPRPSLVALPGPVVAPGANVSLRCAGRIPGMSFALYRVGVATPLQYIDSVQPWA
X1|RAT             LLVTDQLPRPSLVAIPGPVVAPETTVSLRCAGRIPGMSFALYRADVATPLQYIDSVQPWA
                   ****::** *:***:*****.* :.********  .***.*** . *:*:**  * :***

M1|HUMAN           DFTLLGARAPGTYSCYYHTPSAPYVLSQRSEVLVISWEDSGSSDYTRGNLVRLGLAGLVL
M1|CHIMPANZEE      DFTLLGARAPGTYSCYYHTPSAPYVLSQRSEVLVISWEDSGSSDYTRGNLVRLGLAGLVL
X2|HORSE           DFPLLGASAAGTYSCYYHTPSSPYVLSRRSEPLVISLEGSGSLDYTRGNLIRLGLAGLVL
X1|PIG             DFPLPGARAPGTYSCYYHTPSAPYVLSQRSEPLVISADGSGSSDYTQGNVVRLGLAGLVL
X1|DOG             DFPLPGARAPGTYSCYYHTPSSPYVLSLRSEPLVISADGSGSLDYTQSNLVRLGLAGLVL
M2|MOUSE           DFLLIGTHTPGTYCCYYHTPSAPYVLSQRSQPLVISFEGSGSLDYTQGNLIRLGLAGMVL
X1|RAT             DFLLNSANAPGTYYCYYHTPSSPYVLSERSQPLVISSEGSGSLDYTQGNLVRLGLAGLVL
                   ** * .: :.*** *******:***** **: **** :.*** ***:.*::******:**

M1|HUMAN           ISLGALVTFDWRSQNRAPAGIRP-----
M1|CHIMPANZEE      ISLGALVTFDWRSQNRAPAGIRP-----
X2|HORSE           ISLGTLVVFDWCSQSPALGSV-------
X1|PIG             AFLGTLVVFDWRSRSRAPGSMWA-----
X1|DOG             ISLGTLVVFDWHSQSRTQSVRP------
M2|MOUSE           ICLGIIVTCDWHSRSSAFDGLLPQQN--
X1|RAT             ICLGIIVTFDWHSRRSAFVRLLPQQNWV
                     ** :*. ** *:  :
```

Supplementary Figure 3. Sequences of representative OSCAR mammalian orthologues. Multiple alignment of OSCAR sequences of several organisms. The isoform closer to human OSCAR-M1 has been chosen in each case. Database entry codes for each sequence are given in Table 1. Sequences for the predicted signal peptide, the extracellular domains D1, D2 and the predicted TM region are color-coded as in Supplementary Figure 2. A conserved arginine residue in the TM region is highlighted in yellow. Asterisks (*) indicate a fully conserved position; colons (:) and dots (.) indicate a partially conserved position.
